# Physiological and transcriptomic analysis of yellow leaf coloration in *Populus deltoides* Marsh

**DOI:** 10.1371/journal.pone.0216879

**Published:** 2019-05-21

**Authors:** Shuzhen Zhang, Xiaolu Wu, Jie Cui, Fan Zhang, Xueqin Wan, Qinglin Liu, Yu Zhong, Tiantian Lin

**Affiliations:** 1 College of Landscape Architecture of Sichuan Agricultural University, Chengdu, Sichuan, China; 2 College of Forestry of Sichuan Agricultural University, Chengdu, Sichuan, China; VIT University, INDIA

## Abstract

*Populus deltoides* Marsh has high ornamental value because its leaves remain yellow during the non-dormant period. However, little is known about the regulatory mechanism of leaf coloration in *P*. *deltoides* Marsh. Thus, we analyzed the physiological and transcriptional differences of yellow leaves (mutant) and green leaves (wild-type) of *P*. *deltoides* Marsh. Physiological experiments showed that the contents of chlorophyll (Chl) and carotenoid were lower in mutant leaves, and the flavonoid content did not differ significantly between mutant and wild-type leaves. Transcriptomic sequencing was further used to identify 153 differentially expressed genes (DEGs). Functional classifications based on Gene Ontology enrichment and Genome enrichment analysis indicated that the DEGs were involved in Chl biosynthesis and flavonoid biosynthesis pathways. Among these, geranylgeranyl diphosphate (CHLP) genes associated with Chl biosynthesis showed down-regulation, while chlorophyllase (CLH) genes associated with Chl degradation were up-regulated in yellow leaves. The expression levels of these genes were further confirmed using quantitative real-time PCR (RT-qPCR). Furthermore, the estimation of the main precursors of Chl confirmed that CHLP is a vital enzyme for the yellow leaf color phenotype. Consequently, the formation of yellow leaf color is due to the disruption of Chl synthesis or catabolism rather than flavonoid synthesis. These results contribute to our understanding of mechanisms and regulation of leaf color variation in poplar at the transcriptional level.

## Introduction

Leaf color is an important feature of ornamental plants, and trees with colored leaves have been widely cultivated in landscape gardens. The main factors that determine foliage color are the pigment types and their relative concentrations. The formation of red leaves is the result of anthocyanin accumulation, which has been extensively studied [1]. In contrast, there are only a few studies focused on the mechanism of yellow leaves. Leaf yellowing is generally considered to be caused by decreased chlorophyll (Chl) content because Chl is the main pigment content of green leaves [2]. Therefore, studies of leaf yellowing have mostly focused on Chl biosynthesis and degradation. In addition, leaf yellowing may be due also to the accumulation of flavonoids, such as flavanol, flavonol, chalcone and aurone [3,4].

The Chl biosynthetic pathway consists of about 20 different enzymatic steps, starting from glutamyl-tRNA to Chl a and Chl b [5]. Mutations in any one of the genes of the pathway can affect the accumulation of Chl [6], decrease photosynthetic capacity [7] and affect the development of chloroplast [8]. The silence of *CHLD* and *CHLI* (magnesium chelatase subunit D and I) induced by viruses in peas resulted in yellow leaf phenotypes with rapid reduction of photosynthetic proteins, undeveloped thylakoid membranes, altered chloroplast nucleoid structure and malformed antenna complexes [9]. Moreover, in rice, *PGL*_*10*_ encoded protochlorophyllide oxidoreductase B (PORB), pale-green leaf mutant pgl_10_ had decreased Chl (a and b), carotenoid contents, as well as grana lamellae of chloroplasts compared with the wild-type [10]. In addition, mutants with disrupted Chl degradation were used to characterize many steps in the Chl degradation pathway in leaves undergoing senescence [11]. In *Arabidopsis* mutants deficient in PPH (pheophytinase), Chl degradation is inhibited and the plants exhibit a type C stay-green phenotype during senescence [12]. Previous studies revealed that chlorophyllase (Chlase) is involved in Chl degradation in ethylene-treated citrus fruit and could regulate the balance between different plant defense pathways and enhance plant resistance to bacteria [13–15]. Recently, mutants deficient in Chl biosynthesis and degradation have been identified in many yellow leafed plants, such as rice [16–19], *Arabidopsis thaliana* [20] and pak-choi [21].

**Fig 1:**
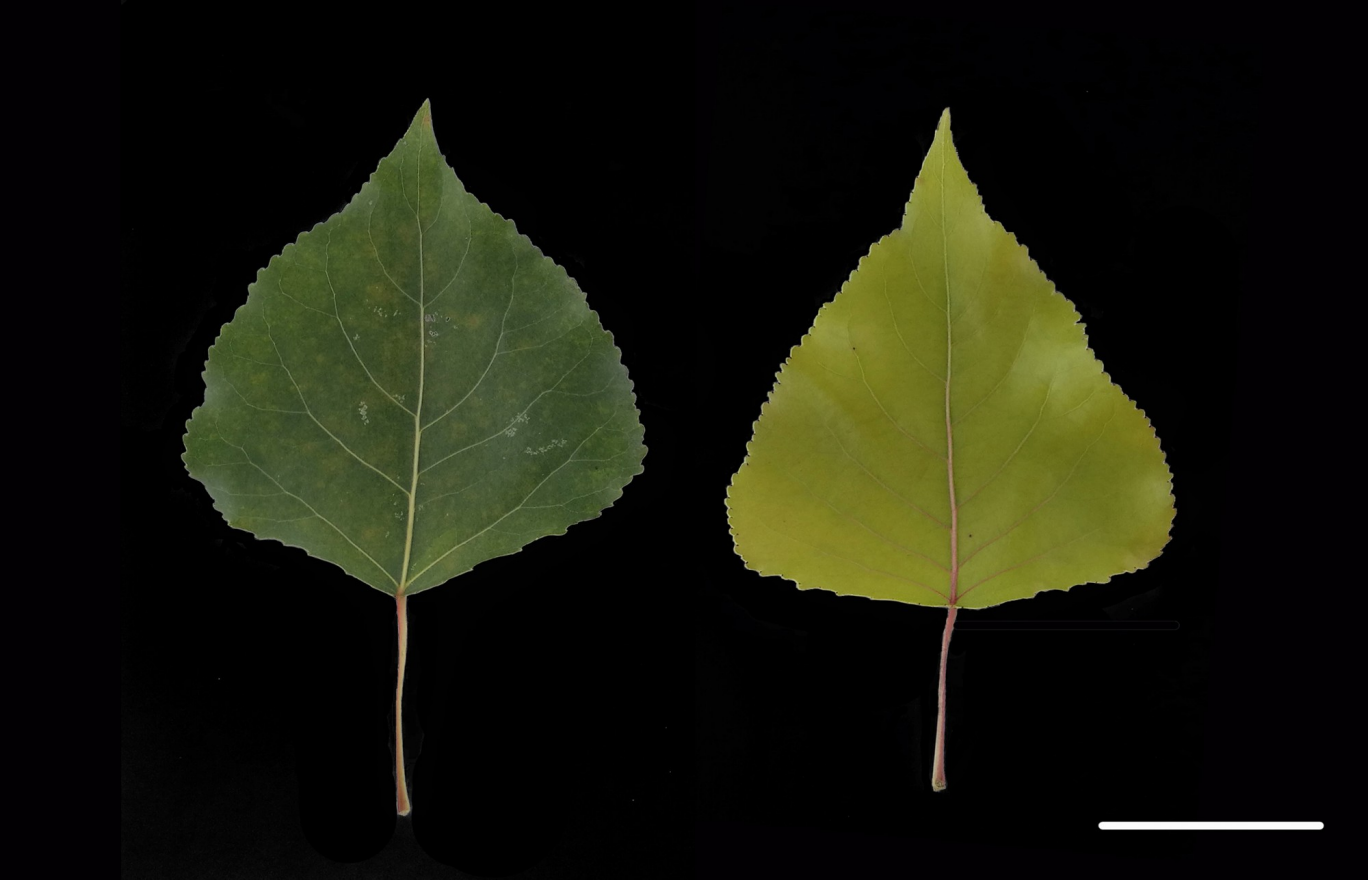
Wild-type (left) and mutant (right) leaves phenotype. Bar = 5 cm.

The genotype *P*. *deltoides* Marsh (mutant) is a bud mutation of green leaf *P*. *deltoides* Marsh (wild-type) (Fig 1). The mutant is a rare deciduous yellow leaf variety in poplar plants of the Salicaceae family. This species has extremely high ornamental value because its leaves remain golden in spring, summer and autumn. However, the molecular mechanism underlying the leaf color of the mutant has not yet been elucidated. Bud mutation is a type of somatic mutation resulting in visible differences from the remainder of the plant in size, shape, color of fruit, branch and flower. Bud mutation arises from genetic mutation of the bud meristem cell [22]. Many ornamental plant cultivars with fruit or flower color variation arose from the bud mutation. For instance, the color in grape skin changes from white to red due to bud mutation [23]. Flower color mutants of roses, carnations and chrysanthemums have also been reported [24]. In contrast, yellow leaf phenotype caused by bud mutations were hardly reported, but the studies related to yellow leaf color were mostly focused on leaf yellowing. For example, the tea cultivar ‘Anji Baicha’ produces yellow or white shoots at low temperatures and turns green when the environmental temperature increases [25]. Only a few studies have reported the yellow leaf phenotype, such as the cucumber Chl-deficient golden leaf mutation [26].

In this study, the photosynthetic pigments, Chl precursors, and flavonoids were estimated and transcriptome level changes of the mutant-type and wild-type were analyzed. Based on a combination of physiological analysis and bioinformatics, we identified differentially expressed genes (DEGs) related to Chl and flavonoid biosynthesis. Furthermore, the expression of DEGs involved in leaf coloration was validated using quantitative real-time polymerase chain reaction (RT-qPCR). Our results clarified the physiological and transcriptomic aspects of golden leaf coloration in *P*. *deltoides* Marsh and will serve as a platform to advance the understanding of the regulatory mechanisms underlying the leaf color formation in poplar and other plant species.

## Materials and methods

### Plant materials

The green leaf *Populus* cultivar (wild-type) and the yellow leaf *Populus* cultivar (mutant) were used in this study. The plants were three-years-old and grown in Hongxia Nursery, Mianzhu Town, Sichuan Province, China. Leaf tissues were collected in May, sampling three leaves per plant for five plants of each type. The leaves were frozen immediately in liquid nitrogen after collection and stored at −80°C for subsequent experiments.

### Pigment content estimates

Approximately 0.1 g wild-type and mutant leaves were selected for Chl and carotenoid estimations. The pigment (Chl a, Chl b, and carotenoid) contents were estimated using the method described by Lichtenthaler [27]. Uroporphyrinogen III (Urogen III) and coproporphyrinogen III (Coprogen III) were extracted and determined as described by Bogorad [28]. Leaves were ground into powder with liquid nitrogen and dissolved in nine volumes of phosphate-buffered saline (pH 7.4) in an ice bath and centrifuged (30 min at 8000 rpm) to estimate the contents of protoporphyrin IX (Proto IX), magnesium protoporphyrin IX (Mg-Proto IX), protochlorophyllide (Pchlide) and chlorophyllide (Chlide) a. The supernatant was used to determine pigments using ELISA kit (MEIMIAN, Jiangsu, China) with a Thermo Scientific Multiskan FC (Thermo Fisher Scientific, MA, USA). Flavonoid contents were estimated using a UV1901 PCS Double beam UV-VIS Spectrophotometer (Shanghai Yoke Instrument Co., Ltd., Shanghai, China) according to the instructions of the Favonoid Plant kit (Suzhou Comin Biotechnology Co., Ltd., Jiangsu, China). Three biological replicates were evaluated for each sample. The data were analyzed with t-tests using version 17.0 of SPSS software (SPSS Inc., Chicago, IL, USA), and means were compared at the significance levels 0.01 and 0.05. Wild-type was used as a control and calculated as 1 for the relative values of photosynthetic pigments and Chl precursors.

### Extraction of RNA

Total RNA was isolated from the wild-type and mutant using the CTAB extraction method. RNA concentration and quality were checked with the Agilent 2100 Bioanalyzer (Agilent Technologies, Santa Clara, USA). RNA purity was measured with a Nano Drop 2000 (Thermo Scientific, USA).

### Library preparation for transcriptome sequencing

Two RNA samples were treated with DNaseI to remove any remaining DNA, and then the oligo (dT) magnetic beads were used to collect the poly A mRNA fraction. After mixing the poly A mRNA fraction with fragmentation buffer, the resulting mRNA was broken into short RNA inserts of approximately 200 nt. The fragments were used to synthesize the first cDNA strand via random hexamer priming, and the second-strand cDNA was then synthesized using DNA polymerase I and RNase H. The cDNA fragments were purified using magnetic beads and subjected to end-repair before adding a terminal A at the 3' ends. Finally, sequencing adaptors were ligated to the short fragments, which were purified and amplified via polymerase chain reaction (PCR). The two libraries were generated and then sequenced on an Illumina HiSeqTM 4000 platform by Chengdu Life Baseline Technology Co., Ltd. (Chengdu, China).

### Quality control and mapping of reads

The raw reads were edited to remove adapter sequences, low-quality reads, and reads with >10% of Q < 20 bases, and then mapped using HISAT v2.0.0 software (http://ccb.jhu.edu/software/hisat2/downloads/) to the *Populus trichocarpa* Torr. & Gray genome. All the clean reads are available at the National Center for Biotechnology Information (NCBI) Short Read Archive (SRA) Sequence Database (accession number SRA740964).

### Gene expression analysis

For gene expression analysis, gene abundance was estimated by RSEM v1.2.30 (http://dewe/ylab.github.io/RSEM/) and then normalized with fragments per kilobase of exon per million mapped reads (FPKM) values [29]. The NOIseq v2.16.0 (http://www.bioconductor.org/packages/release/bioc/html/NOISeq.html) was used to identify genes that were differently expressed between wild-type and mutant in this experiment. Genes with probability>0.8 and |log2 fold change| ≥ 1 were considered as DEGs between samples.

For functional annotation, GO enrichment analysis of DEGs was performed in the GO database (http://www.geneontology.org/) to calculate gene numbers for every term. The hypergeometric test was conducted to find significantly enriched GO terms in the input list of DEGs. KEGG enrichment analysis was implemented using the database resource (http://www.genome.jp/kegg/). The calculation method of KEGG analysis is the same as the GO analysis.

### Real-time RT-PCR

For qPCR analysis, total RNA was extracted using the RNAprep Pure Plant Kit (Tiangen Biotech Co. Ltd., Beijing, China). Approximately 1 μg RNA was reverse transcribed via a TransScript All-in-One First-Strand cDNA Synthesis SuperMix for qPCR (Tiangen Biotech Co. Ltd., Beijing, China) according to the manufacturer's instructions. Eight genes were selected for validation using qRT-PCR. Primer sequences were designed using Primer Premier 5.0 software as shown in S3 Table. qPCR DNA amplification and analysis were performed using the TransScript Top Green qPCR SuperMix kit (Tiangen Biotech Co. Ltd., Beijing, China) in accordance with the manufacturer’s protocol with an CFX Connect Real-Time System (Bio-Rad, Hercules, CA, USA). The thermal profile was as follows: pre-denaturation at 94°C for 30 s; 94°C for 5 s, 60°C for 30 s, for 40 cycles. The relative expression level of selected genes in wild-type and mutant was normalized to CDC2 and ACT expression. Three biological replicates for each of the reactions were performed. The relative expression levels of target genes were estimated using the 2^–ΔΔCt^ method [30].

## Results

### Pigment content analysis

**Fig 2:**
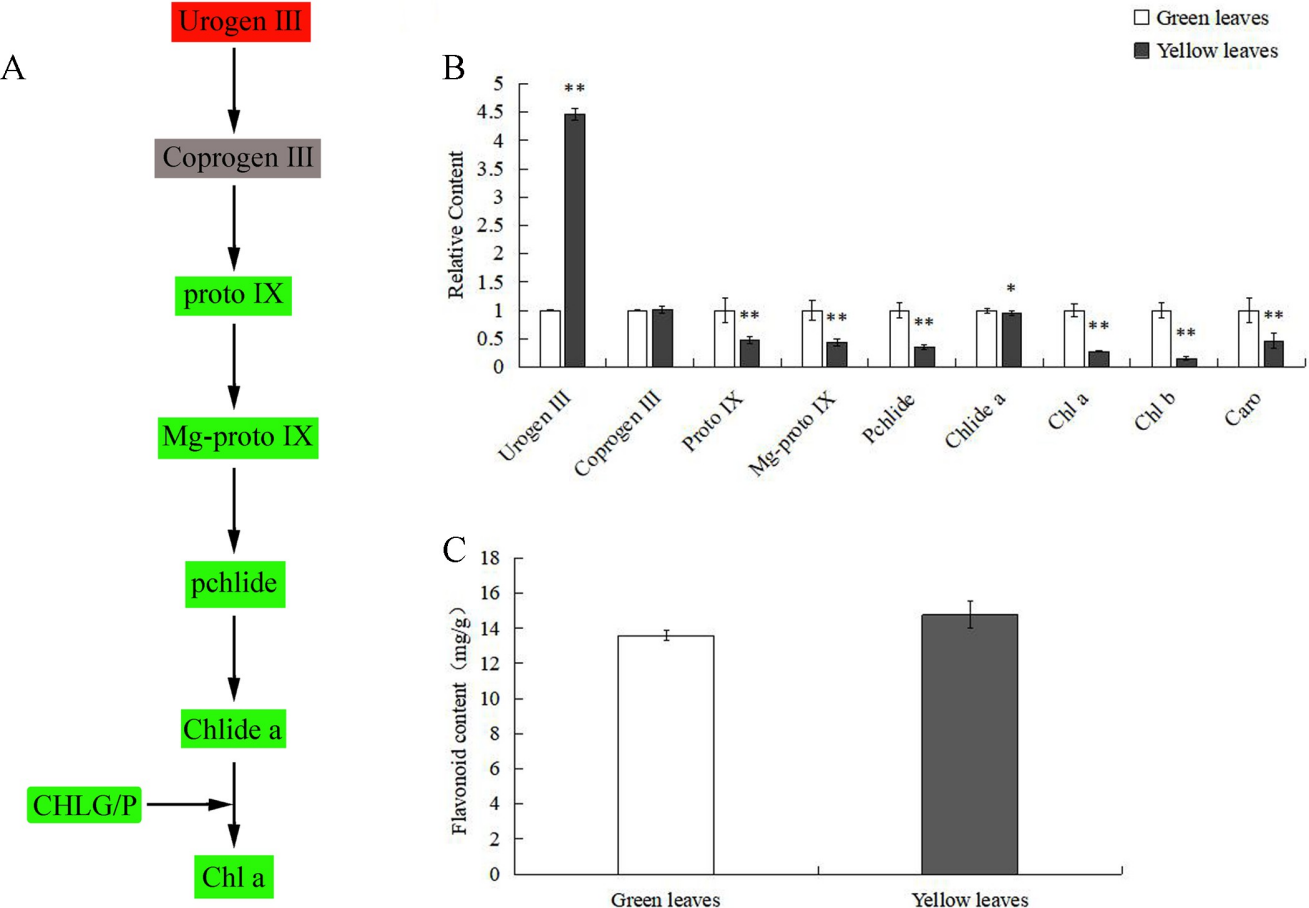
Pigment contents in wild-type and mutant leaves. (A) Schematic view of the Chl biosynthetic pathway. Urogen III (in red) at the beginning of the pathway shows a significant increase in yellow leaves. CHLG/P illustrates the gene encoding protein catalyzing the reaction of the precursors. A green box represents each component that is significantly decreased or down-regulated in the yellow leaves. Coprogen III had no significant difference between green and yellow leaves. (B) Comparison of the relative content of Chl precursors and photosynthetic pigments between wild-type and mutant. (C) Comparison of the flavonoid contents between wild-type and mutant leaves. Asterisks indicate: (*) P ⩽ 0.05, (**) P ⩽ 0.01. Abbreviations are: Urogen III = uroporphyrinogen III; Coprogen III = coproporphyrinogen III; Proto IX = protoporphyrin IX; Mg-Proto IX = Mg-protoporphyrin IX; Pchlide = protochlorophyllide; Chlide a = chlorophyllide a; Chl a = chlorophyll a; Chl b = chlorophyll b; and Caro = carotenoid.

We analyzed changes in the pigment contents of wild-type and mutant leaves. The Urogen III content of the yellow leaves was significantly higher than that of the wild-type, whereas there were no significant differences in Coprogen III (Fig 2). Proto IX, Mg-Proto IX and Pchlide contents of the mutant were significantly decreased by about 52.53%-64.71% than green leaves. The content of Chlide a in yellow leaves was lower than that of green leaves. Compared with the green leaves, the Chl a content, Chl b content and carotenoids content of yellow leaves were significantly lower by 72.41%, 84.86% and 53.88%, respectively (Fig 2). The ratio of carotenoids to chlorophylls in yellow leaves was 0.12, which was higher than that of green leaves (0.06). In addition, the difference between the total flavonoid contents of the green leaves and yellow leaves was not significant (Fig 2).

### RNA-seq analysis

RNA-seq libraries were constructed from green and yellow leaf samples and sequenced using the Illumina HiseqTM 4000 platform for acquiring a comprehensive overview of leaf coloration. Approximately 45 million and 47 million raw reads were obtained from green and yellow leaves, respectively. After removal of adapter sequence and low-quality reads, the number of clean reads in the two libraries was 40,779,290 and 41,776,346. The Q20 and Q30 of the two samples were at least 97.28 and 93.20%, respectively, and the GC content of both exceeded 45%. Additionally, 73.79% of green leaves and 71.67% of yellow leaves reads of each sample were mapped to the *Populus trichocarpa* Torr. & Gray genome sequence and approximately 47% of the mapped reads were found to be unique (Table 1).

**Table 1:** Summary of the sequencing and mapping results.

Sample name	Green leaves	Yellow leaves
Raw reads	45846322	47483602
Clean reads	40779290	41776346
Q20(%)	97.45	97.28
Q30(%)	93.57	93.20
GC content(%)	45.36	45.14
Total mapped	30092762 (73.79%)	29942722 (71.67%)
Uniquely mapped	9961342 (48.85%)	9903152 (47.41%)

### Gene expression analysis

**Fig 3:**
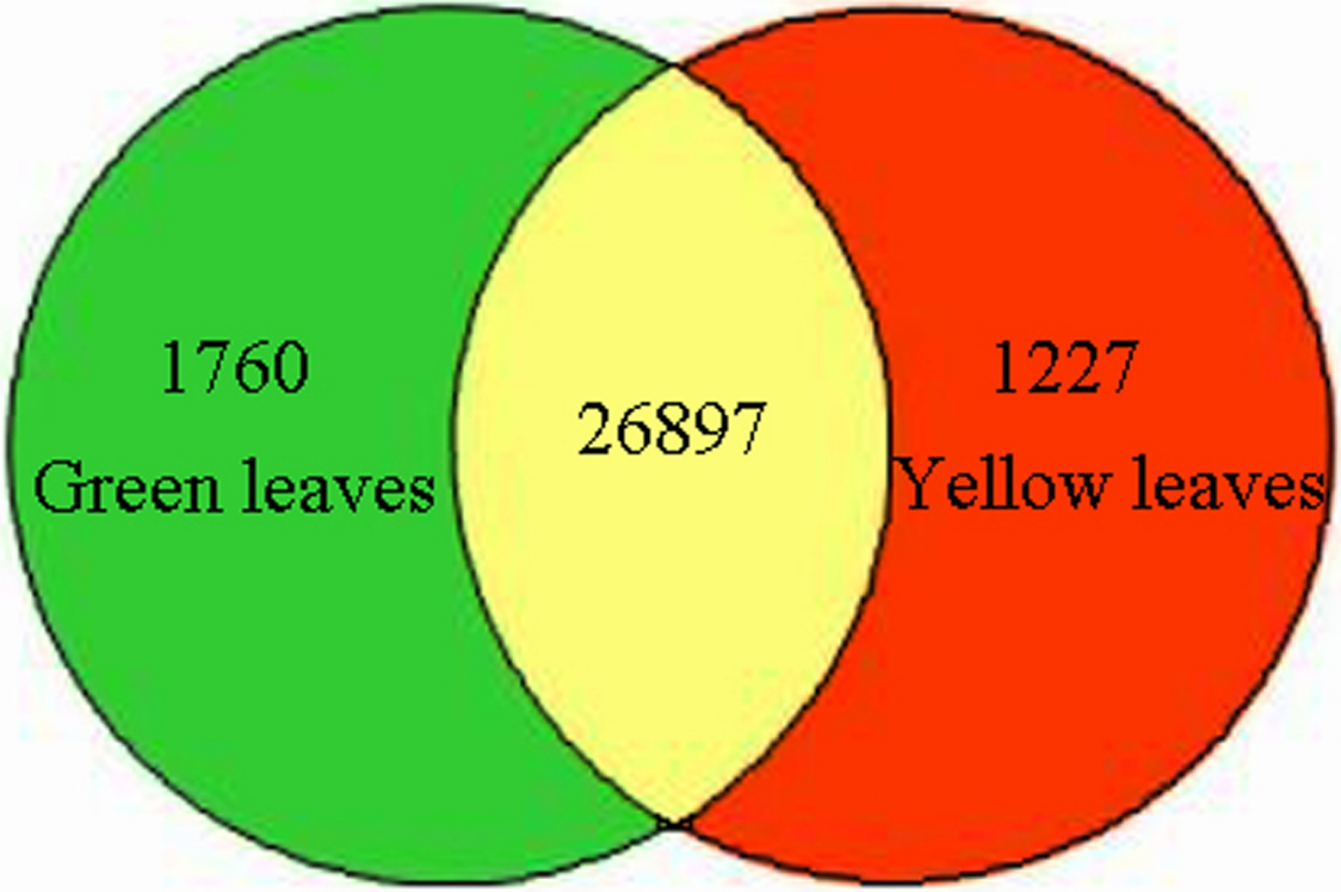
Number of specific and shared genes between wild-type and mutant leaves.

In total, the number of expressed genes were 28,657 and 28,124 in green and yellow leaves, respectively, of which 1760 and 1227 genes were expressed specifically in the green and yellow leaves (Fig 3). In order to identify DEGs between green and yellow leaves, we set the expression of genes in green leaves as the control and identified genes that were up- or downregulated in yellow leaves. Accordingly, a total of 153 DEGs were found in yellow leaves, including 52 up-regulated genes and 101 down-regulated genes.

### Gene functional annotation

**Fig 4:**
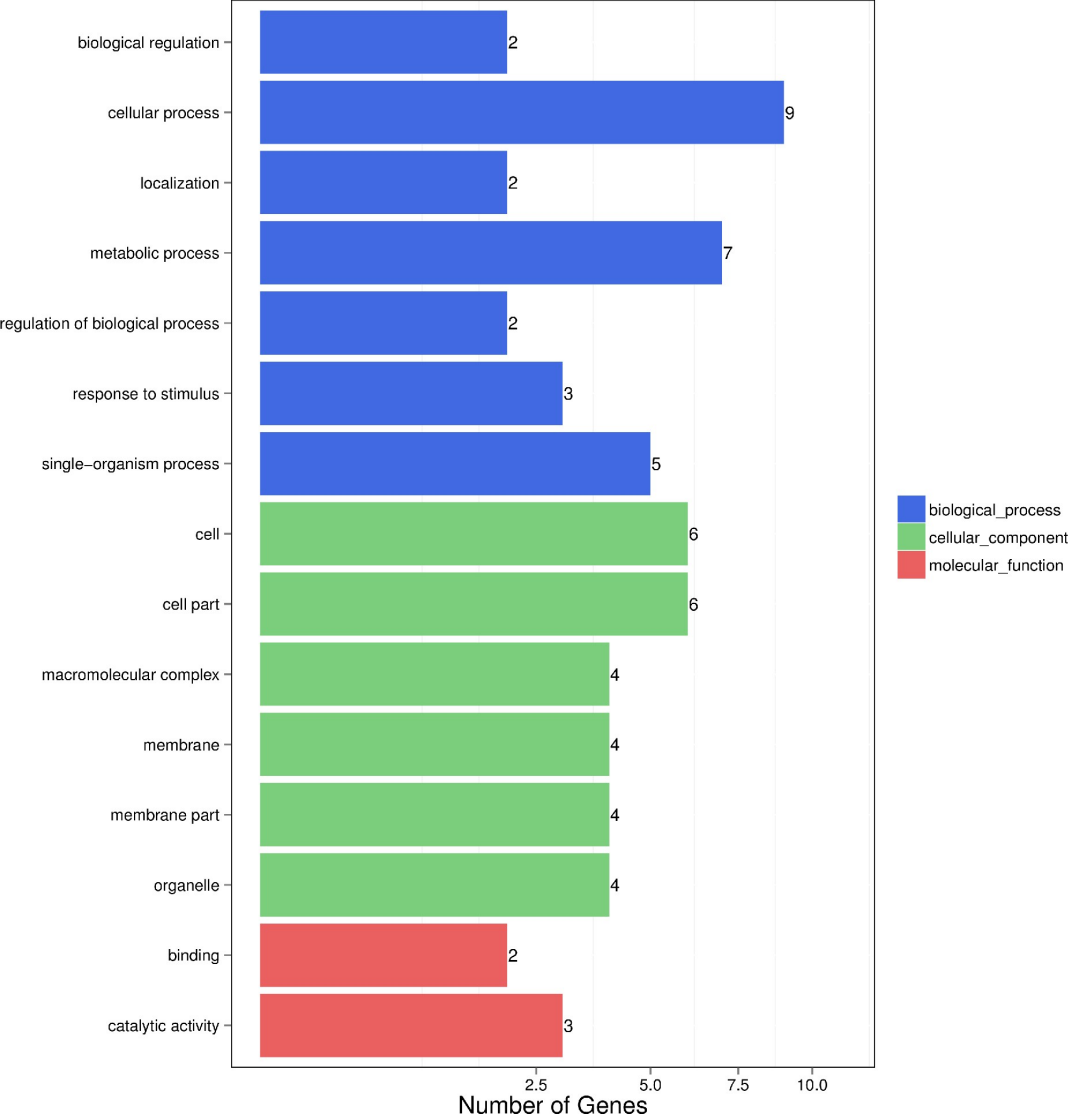
The DEG function classification in wild-type and mutant leaves. X-axis displays the number of genes. Y-axis is broken into three categories: biological process, cellular component and molecular function with enrichment details within each category.

GO assignments were used to classify the functions of DEGs. A total of 12, 9, and 5 of the DEGs were divided into biological processes, cellular components and molecular functions, respectively, and some DEGs were annotated with more than one GO term (Fig 4). In the biological process category, many DEGs fell into the categories of ‘cellular process,’ ‘metabolic process,’ and ‘single-organism process’ (S1 Table). The most enriched terms of the cellular component were involved in ‘cell,’ ‘cell part,’ ‘membrane’ and ‘membrane part’ were also significantly enriched terms (S1 Table). Meanwhile, the dominant categories with respect to molecular function group were ‘binding’ and ‘catalytic activity’ (S1 Table).

**Fig 5:**
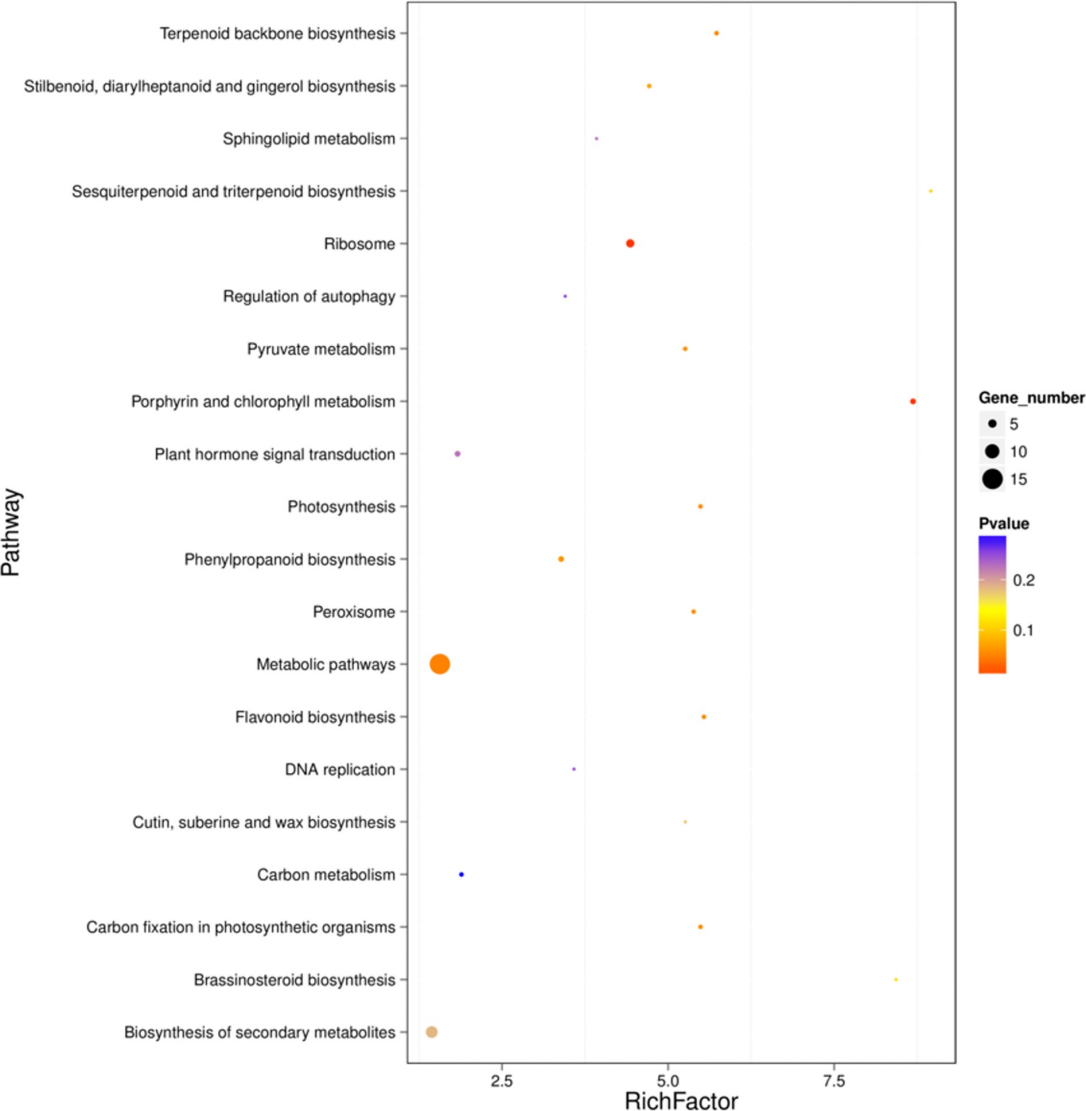
Enriched KEGG pathways of DEGs. X-axis displays the Rich factor. Y-axis names the KEGG pathways. Dot size corresponds to the number of DEGs in the pathway with smaller dots having a lower gene number than larger dots. Dot color indicates P value with red having a high P value and blue having a low P value.

KEGG pathway analysis was performed to categorize gene functions with an emphasis on biochemical pathways that were active in green and yellow leaves. A total of 52 genes were annotated and assigned to 31 KEGG pathways (S2 Table). The most significantly enriched pathway was ‘Metabolic pathways’ (Fig 5), with 15 associated DEGs (ranked by padj value), followed by ‘Biosynthesis of secondary metabolites’ and ‘Ribosome’ with 8 and 5 DEGs, respectively, which supported the results of GO assignments that ‘metabolic process’ was significantly enriched. Moreover, 3 DEGs were assigned to ‘Porphyrin and Chl metabolism’ and 2 DEGs were assigned to ‘Flavonoid biosynthesis.’ This cluster of results indicated that the differences in metabolic activities were the main difference between green and yellow leaves, and they may perform important roles in the regulating of leaf coloration.

### Chl and flavonoid biosynthesis analysis

**Fig 6:**
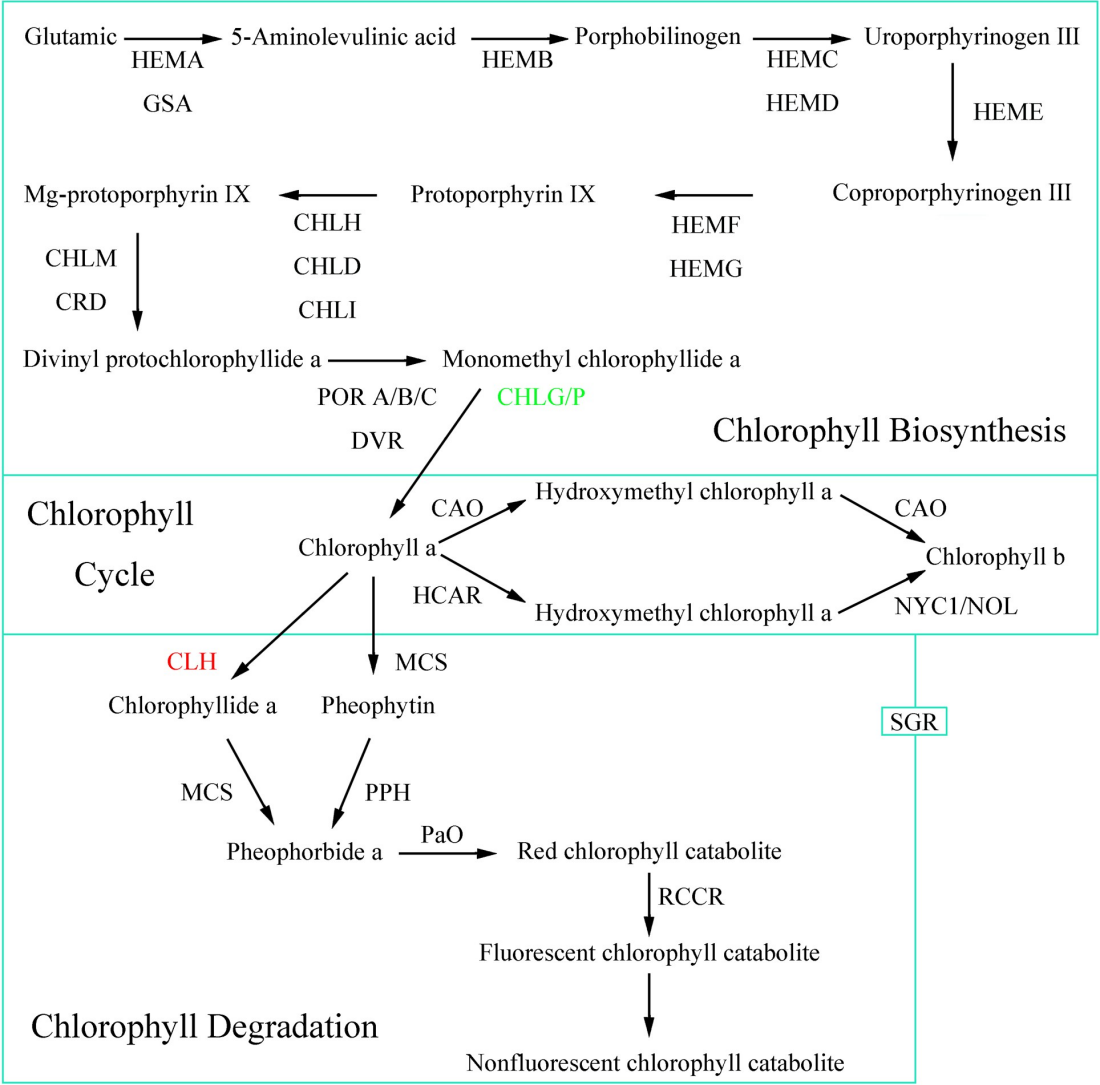
Chl biosynthetic pathways at the transcript level. Red notates up-regulated genes and green marks down-regulated genes.

Based on the above annotations, we found that the *P*. *deltoides* Marsh transcriptome contains genes involved in Chl biosynthesis and flavonoid biosynthesis (Table 2). Two genes annotated as CHLP (*Potri*.*019G009000* and *Potri*.*019G024600*) were down-regulated in yellow leaves. In the last step of Chl a biosynthesis, the geranylgeranyl diphosphate (CHLP, EC:1.3.1.111) catalyzed the reduction of geranylgeranyl pyrophosphate to phytyl pyrophosphate and yielded Chl (Fig 6). Furthermore, the gene encoding Chlase (CLH, EC:3.1.1.14) played roles in the transition of Chl a(b) to Chlide a(b), which was found to be up-regulated in yellow leaves. In flavonoid biosynthesis, two genes annotated as shikimate O-hydroxycinnamoyltransferase (HCT, EC:2.3.1.133) were differentially expressed in green and yellow leaves. Of these, one gene (*Potri*.*006G034100*) was more highly expressed in green leaves while the other gene (*Potri*.*005G028500*) was more highly expressed in yellow leaves.

**Table 2:** DEGs involved in Chl and flavonoid biosynthesis in mutant transcriptome.

Function	Gene ID	Seq. Description	log2FC
chlorophyll biosynthesis	Potri.019G009000	GDSL-like Lipase/Acylhydrolase superfamily protein	-10.7764
	Potri.019G024600	GDSL-like Lipase/Acylhydrolase superfamily protein	-10.1319
	Potri.005G214100	chlorophyllase 1	9.6073
flavonoid biosynthesis	Potri.005G028500	HXXXD-type acyl-transferase family protein	9.2808
	Potri.006G034100	HXXXD-type acyl-transferase family protein	-9.8978

### Validation of RNA sequencing data

**Fig 7:**
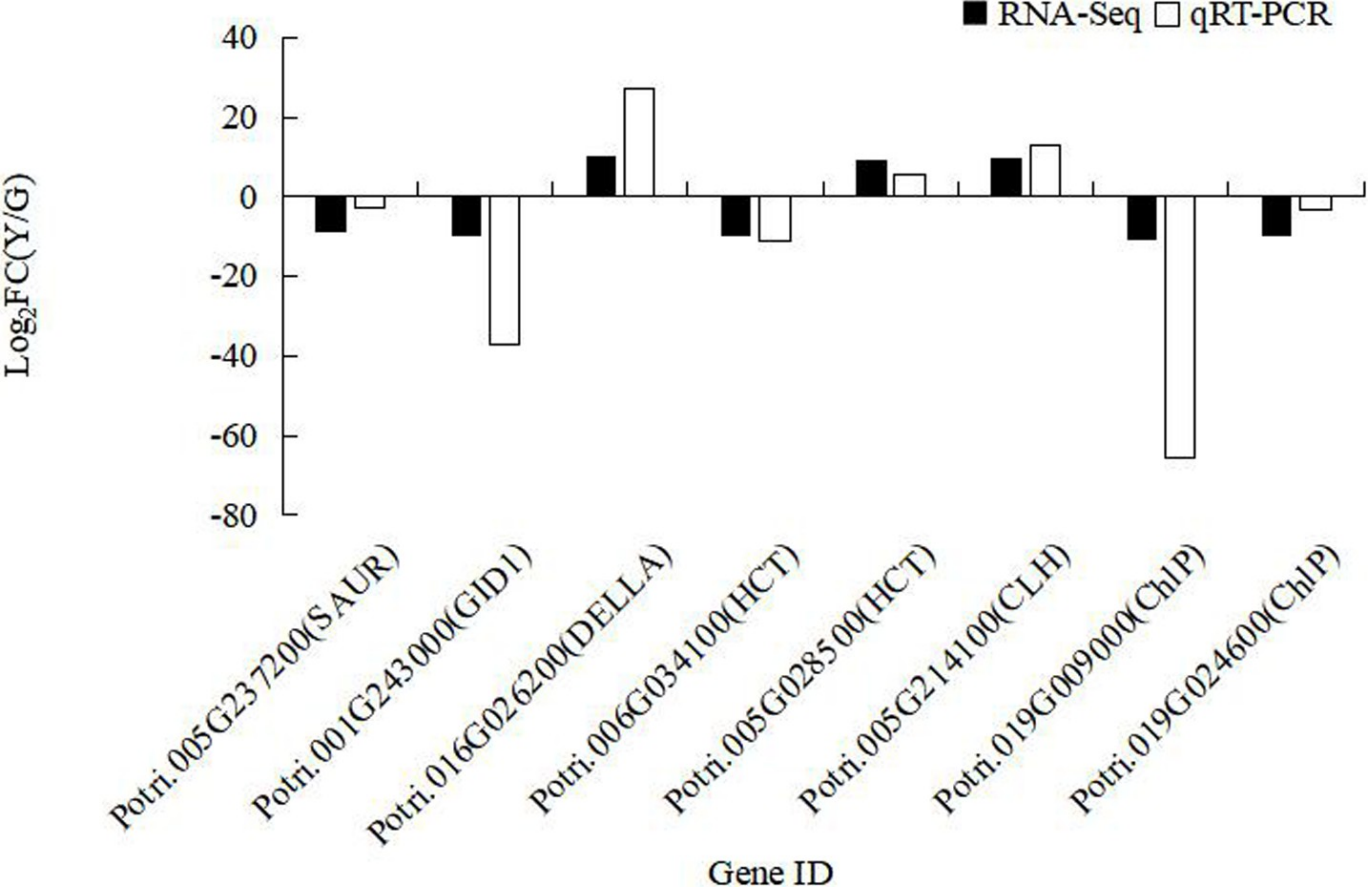
RT-qPCR vs. RNA-seq analysis of 8 DEGs in mutant leaves. X-axis is the Gene ID. Y-axis is the fold change in mutant leaves relative to that in wild-type leaves (Log2(FC)).

To validate the accuracy of RNA-seq expression results, 8 DEGs with marked changes in plant hormone signal transduction, flavonoid biosynthesis and Chl biosynthesis were detected by qPCR (Fig 7). The results showed that except 3 genes (*Potri*.*016G026200*, *Potri*.*005G028500*, *Potri*.*005G214100*), the remaining 5 genes were all down-regulated in mutant plants. In general, qRT-PCR results concur with the RNA-seq data, indicating that the DEGs identified by RNA-seq were accurate.

## Discussion

*P*. *deltoides* Marsh developed yellow leaves during growth and throughout the lifespan by bud mutation. Understanding the regulatory mechanisms underlying the leaf color of *P*. *deltoides* Marsh is of significant importance. Changes in ratio of Chl, carotenoids and flavonoids in leaves will change the expression of leaf color. In this study, the compounds affecting the pigment of leaves were determined. The Chl content in the mutant *P*. *deltoides* Marshwas significantly lower than that of wild-type. Consistent with the physiological results, transcriptional analysis demonstrated that down-regulation of *CHLP* and up-regulation of *CLH* involved in porphyrin and Chl metabolism pathways were crucial genes that resulted in yellow leaves.

Carotenoids provide yellow, orange and red color to flowers and fruits, which play crucial roles in photosystem assembly, light-harvesting and photoprotection [31,32]. In this study, the carotenoid content decreased significantly in mutant compared with wild-type leaves. Significant reductions in the carotenoid content were also observed in previous studies in yellow-green winter wheat mutant *Ygm* and Pak-choi yellow leaf mutant *pylm* [33, 21]. However, in *Ginkgo biloba* L, the carotenoids content in the mutant leaves was higher than that of normal green leaves [2]. This carotenoid content may have been higher because the mutants of *Ginkgo biloba* were special golden–green striped leaves. The ratio of carotenoids to Chl drives yellow leaf coloration, and the photosynthetic pigment contents in the green leaves are similar to the yellow parts of mutant leaves. By contrast, the mutant in our study is normal, and the total photosynthetic pigment contents in green leaves are 4 times that of yellow leaves. In addition, increased ratio of carotenoids to Chl was observed in both *pylm* mutant and *Ginkgo biloba* mutant [21, 2]. Consistent with these research findings, the ratio of carotenoids to chlorophylls in yellow leaves was two-fold higher than that in green leaves in our study, thus suggesting that the increase of ratio of carotenoids to chlorophylls was related to yellow coloration in *P*. *deltoides* Marsh. Our transcriptional results demonstrated that no genes were annotated to the carotenoid biosynthesis pathway.

### Chl biosynthetic genes differentially express in leaf color mutants

Leaf color formation is closely related to Chl biosynthesis and breakdown, and most leaf color mutations are Chl-deficiency mutations [34]. Chl is responsible for harvesting solar energy and electron transport, even turning plants green because it is Mg^2+^-containing tetrapyrrole pigment [35]. In this study, the novel Chl-deficient chlorina mutant of *P*. *deltoides* Marsh with yellow leaf phenotype was identified. Compared with wild-type, the content of photosynthetic pigments in the mutant were significantly lower. In particular, the Chl b content was six times higher in green leaves than yellow leaves. These results suggest that the yellow leaf phenotype is a result of a lack of Chls.

The Chl metabolic process can be subdivided into three parts: biosynthesis of Chl a, the Chl cycle between Chl a and b, and degradation of Chl a [36–48]. Chl is composed of two moieties, Chlide and phytol, which are respectively formed from the precursor molecules 5-aminolevulinate and isopentenyl diphosphate [39]. *CHLP* encodes the enzyme geranylgeranyl reductase catalyzing terminal hydrogenation of geranylgeraniol to phytol for Chl synthesis [40,41]. Previous studies revealed that in transgenic tobacco (*Nicotiana tabacum*) expressing antisense *CHLP* RNA, transformants with gradually reduced *CHLP* expression displayed a uniform low pigmentation and a pale or variegated phenotype [42]. In cyanobacterium *Synechocystis* sp. PCC 6803, *ΔchlP* mutant exhibit decreased Chl and total carotenoids contents, and unstable photosystems I and II [43]. Two *CHLP* genes (Potri.019G009000 and Potri.019G024600) were identified in our database and both were down-regulated in the mutant. In the meantime, qPCR experiments further verified that expression levels of *CHLP* genes in mutant were highly reduced compared to those in wild-type, which suggests a later stage of Chl biosynthesis was inhibited. Parallel experiments also showed that the content of Chlide a was about 4.83% lower, while the content of Chl a was 72.41% lower in the yellow leaves compared to green leaves. The result suggests that the inhibition of enzyme activity of CHLP protein is likely to further suppress the biosynthesis of Chl in yellow leaves. In addition, our physiological results show that the content of Urogen Ⅲ in the yellow leaves is about 4 times than that of the green leaves, but the content of Coprogen III is not significantly different between green and yellow leaves. Therefore, there might be a suppression between Urogen Ⅲ and Coprogen III during Chl biosynthesis. However, the results need further verification.

Chl breakdown starts with the reduction of Chl b, which is then converted into Chl a via two steps of enzymatic reaction, and finishes by producing nonfluorescent chlorophyll catabolite or nonfluorescent dioxobilin-type chlorophyll catabolite [44]. Chlase, the Chl dephytilation enzyme, catalyzes the hydrolysis ester bond of Chl to yield Chlide and phytol [45]. Chlase activity is negatively correlated with Chl levels of color break in citrus fruits and Chlase participates in the Chl breakdown in citrus [15,46,47]. The citrus *Chlase1* gene was overexpressed in squash and tobacco. Expression of Chlase without the N-terminal 21 amino acids (ChlaseΔN) resulted in chlorosis in plants, whereas expression of the fulllength Chlase resulted in moderate Chl breakdown [46]. Subsequently, citrus Chlase was demonstrated to be translated and processed to a mature form, which is subject to dual N- and C-terminal processing [15,47]. However, some evidence does not support that Chlase play a critical role in Chl degradation during leaf senescence [48–51]. For example, overexpression of *ATHCOR1*, which has Chlase activity in *Arabidopsis*, led to an increased breakdown of Chl a, but the total Chl level was not affected [48]. Similarly, a short delay in yellowing was observed in the antisense *BoCLH1*-positive transformants [49]. *Arabidopsis* Chlases (*AtCLH1* and *AtCLH2*) are not positively regulated with leaf senescence. *CHL1* and *CHL2* single and double knockout mutant plants do not display a significant delay in senescence [50]. A previous study suggested that Chlase was not involved in Chl breakdown in the absence of methyljasmonate and exhibited a defense response when plants were damaged [51]. Schelbert et al. also supported the opinions that Chlase was not to be essential for dephytylation after Chl is converted into pheophorbide [12]. In our study, the transcript expression patterns suggested that the expression of *CLH* was higher in wild-type than in mutant. Moreover, previous studies in common wheat (*Triticum aestivum* L.) showed that the gene encoding Chlase in the Chl biosynthesis pathway was also significantly up-regulated in the yellow leaf mutant [33]. Therefore, experiments related to cloning and functional verification of *CLH* in *P*. *deltoides* Marsh are needed to further verify the function of Chlase in Chl breakdown.

### Flavonoid biosynthetic genes differentially express in leaf color mutants

Flavonoids, carotenoids, and Chls are the main pigments responsible for flower and leaf color. Previous studies have demonstrated that flavonoids are the main pigments, producing purple, blue, yellow, and red colors in plants [52]. Flavonoids have been known as UV-protecting pigments and antioxidants by scavenging molecular species of active oxygen [53,54]. In *Ficus microcarpa* L. f., the golden leaf mutant is the result of continuous high-light irradiation, and the flavonoid level of golden leaf was 5-fold higher than that of green leaf, the results suggest that the increase of flavonoids in the golden leaf may protect the leaves from high-light stress [55]. In this study, there are no significant differences in the content of flavonoid between wild-type and mutant. Therefore, we consider the yellow leaf phenotype to be caused by genetic factors, not environmental factors. Shikimate/quinate hydroxycinnamoyltransferase (E2.3.1.133, HCT) belongs to the large family of BAHD-like acyltransferases [56]. It is a key enzyme that determines whether 4-coumaroyl CoA is the direct precursor for flavonoid or H-lignin biosynthesis [57]. In Arabidopsis, silencing of the *HCT* gene resulted in severely reduced growth and absence of S lignin [58]. The down-regulation of *HCT* have a dramatic effect on lignin content and composition in alfalfa and poplar [59,60]. Up until now, most studies have focused on the effects of *HCT* on lignin synthesis [61, 62], while only a few studies related *HCT* to flower color or leaf color of plants. It is further proven that the blocked Chl synthesis pathway in mutant-type plants may be the consequence of yellowing of the leaves.

## Conclusions

In this study, physiological and transcriptome sequence analysis showed that there were distinct differences in coloration between green and yellow mutant leaves of *P*. *deltoides* Marsh. Transcriptional sequence analysis identified 5 DEGs that participated in porphyrin and Chl metabolism and flavonoid biosynthesis pathways. Furthermore, RT-qPCR verified that those DEGs were expressed differentially in mutant and wild-type plants. Down-regulation of *CHLP* and up-regulation of *CLH* might cause the difference of leaves. These results provide an excellent platform for future studies to uncover the molecular mechanisms underlying the yellowing phenotype in *P*. *deltoides* Marsh and other closely related species.

## Supporting information

S1 Table Significantly enriched gene ontologies among downregulated or upregulated genes in mutant compared to wild-type. GO enrichment analysis of DEGs was performed in the GO database (http://www.geneontology.org/) to calculate gene numbers for every term. The hypergeometric test was conducted to find significantly enriched GO terms in the input list of DEGs.
(DOCX)

S2 Table Wild-type vs. mutant pathway enrichment. KEGG enrichment analysis was implemented using the database resource (http://www.genome.jp/kegg/). The hypergeometric test was conducted to find significantly enriched KEGG terms in the input list of DEGs. The closer Pvalue is to zero, the more significant the enrichment is. some DEGs were annotated with more than one pathway.
(XLSX)

S3 Table Primers for qPCR analysis. (DOCX)
