# Supplementary material for: Physiological and transcriptomic analysis of yellow leaf coloration in Populus deltoides Marsh
Source: PLoS One. 2019 May 21;14(5):e0216879. doi: 10.1371/journal.pone.0216879 (PMC6529213; doi:10.1371/journal.pone.0216879)
Supplement: S1 Table — GO enrichment analysis of DEGs was performed in the GO database (http://www.geneontology.org/) to calculate gene numbers for every term. The hypergeometric test was conducted to find significantly enriched GO terms in the input list of DEGs. (DOCX) [file pone.0216879.s001.docx]

**Supplementary Table S1** Significantly enriched gene ontologies among downregulated or upregulated genes in mutant compared to wild-type

| **Gene ontology (description and term)*^a^*** | **Differentially expressed genes (DEGs)** | | | |
| --- | --- | --- | --- | --- |
|  | **Total** | **Downregulated** | **Upregulated** | **Pvalue** |
| **Cellular Component** |  |  |  |  |
| cell; GO:0005623 | 6 | 3 | 3 | 0.3134 |
| cell part; GO:0044464 | 6 | 3 | 3 | 0.3134 |
| macromolecular complex; GO:0032991 | 4 | 3 | 1 | 0.2785 |
| membrane; GO:0016020 | 4 | 3 | 1 | 0.7947 |
| membrane part; GO:0044425 | 4 | 3 | 1 | 0.2343 |
| organelle; GO:0043226 | 4 | 3 | 1 | 0.4725 |
| **Molecular function** |  |  |  |  |
| binding; GO:0005488 | 2 | 1 | 1 | 0.9249 |
| catalytic activity; GO:0003824 | 3 | 0 | 3 | 0.4693 |
| **Biological Process** |  |  |  |  |
| biological regulation; GO:0065007 | 2 | 0 | 2 | 0.6051 |
| cellular process; GO:0044763 | 9 | 5 | 4 | 0.1943 |
| localization; GO:0051179 | 2 | 2 | 0 | 0.4797 |
| metabolic process; GO:0008152 | 7 | 4 | 3 | 0.8054 |
| regulation of biological process; GO:0050789 | 2 | 0 | 2 | 0.5992 |
| response to stimulus; GO:0050896 | 3 | 0 | 3 | 0.1159 |
| single-organism process; GO:0044699 | 5 | 3 | 2 | 0.6581 |

*^a^*GO enrichment analysis of DEGs was performed in the GO database (http://www.geneontology.org/) to calculate gene numbers for every term. The hypergeometric test was conducted to find significantly enriched GO terms in the input list of DEGs.

Note: an individual gene might be assigned to more than one GO term.
