# Supplementary material for: Physiological and transcriptomic analysis of yellow leaf coloration in Populus deltoides Marsh
Source: PLoS One. 2019 May 21;14(5):e0216879. doi: 10.1371/journal.pone.0216879 (PMC6529213; doi:10.1371/journal.pone.0216879)
Supplement: S3 Table — (DOCX) [file pone.0216879.s003.docx]

**Spplementary Table S3.** Primers for qPCR analysis

| **Gene ID** | **sequence (5’ – 3’)** | **Description** |
| --- | --- | --- |
| *CDC2-F* | ATTCCCCAAGTGGCCTTCTAAG | Cell division control protein |
| *CDC2-R* | TATTCATGCTCCAAAGCACTCC |  |
| *ACT-F* | GTCTGCGACAATGGAACTGGA | Actin |
| *ACT-R* | CTGGCATACAGGGAAAGGACA |  |
| *Potri.005G237200-F* | AAGGCCTTCCTTTGGACGTT | SAUR-like auxin-responsive protein family(SAUR) |
| *Potri.005G237200-R* | AGCAAAGTCTGAAACTCGGGAC |  |
| *Potri.001G243000-F* | TGGCACCGGAGACCAGGAATC | alpha/beta-Hydrolases superfamily protein(GID1) |
| *Potri.001G243000-R* | CTCCATGAGCAGCCAACCAAGG |  |
| *Potri.016G026200-F* | TGATGTCCGCTCCCAAATCC | DELLA protein(DELLA) |
| *Potri.016G026200-R* | GGGAGGATGAGATTGTGGTTGT |  |
| *Potri.006G034100-F* | TGTTCAGTTGCTGGTGTCGGTTC | HXXXD-type acyl-transferase family protein(HCT) |
| *Potri.006G034100-R* | GTTGGTACGTCCACGCTACTCAC |  |
| *Potri.005G028500-F* | TACCTCCACCACGCATGGCTAG | HXXXD-type acyl-transferase family protein(HCT) |
| *Potri.005G028500-R* | TGCCGAGACCACCTCCACAC |  |
| *Potri.005G214100-F* | CACAGGCTGCCAGAACTCTTGG | chlorophyllase 1(CLH) |
| *Potri.005G214100-R* | ATAACTGCGGTGCGACCACTATG |  |
| *Potri.019G009000-F* | GTGCTCATGCCGCTCCACAG | GDSL-like Lipase/Acylhydrolase superfamily protein(ChlP) |
| *Potri.019G009000-R* | CAAGGTTGTTGTTGTTGCCACTATCC |  |
| *Potri.019G024600-F* | GTCGGCGGGAATGATTTCGT | GDSL-like Lipase/Acylhydrolase superfamily protein(ChlP) |
| *Potri.019G024600-R* | ACGTAATCAGGGAGCCGGAA |  |
